# The Confounding Role of Graft-Versus-Host Disease in Animal Models of Cancer Immunotherapy: A Systematic Review

**DOI:** 10.34172/aim.2024.24

**Published:** 2024-03-01

**Authors:** Hami Ashraf, Mohammad Heydarnejad, Farid Kosari

**Affiliations:** ^1^Digestive Disease Research Institute, Tehran University of Medical Sciences, Tehran, Iran; ^2^Department of Pathology, School of Medicine, Shahid Beheshti University of Medical Sciences, Tehran, Iran; ^3^Department of Pathology, Shariati Hospital, School of Medicine, Tehran University of Medical Sciences, Tehran, Iran

**Keywords:** Animal models, Confounder, Graft-versus-host disease, GVHD, Immunotherapy, Preclinical models

## Abstract

**Background:**

Cancer immunotherapy has emerged as a transformative approach for treating various malignancies, including melanoma, lung cancer, breast cancer, and leukemia. Animal models have been instrumental in elucidating the mechanisms and potential of these therapies. However, graft-versus-host disease (GVHD) is an inherent challenge in these studies, primarily because the introduction of foreign immune cells or tissues often triggers immune responses.

**Methods:**

A detailed systematic search was conducted across various scientific databases, including PubMed, Scopus, Embase, and Web of Science. The search aimed to identify peer-reviewed articles published in English from January 2000 to September 2023. Keywords and phrases used in the search included "Graft-versus-Host Disease", "GVHD", "animal models", "cancer immunotherapy", and combinations thereof. Boolean operators (AND/OR) were employed to refine the search. Finally, 6 articles were included in this systematic review, which is registered on PROSPERO (ID number CRD42024488544).

**Results:**

Our systematic review identified several mechanisms employed in animal studies to mitigate the confounding effects of GVHD. These included genetically modified mouse models, immunosuppressive drugs, and humanized mice. Furthermore, the review highlights innovative approaches such as selective T-cell depletion and the use of specific cytokine inhibitors.

**Conclusion:**

By systematically identifying and mitigating the confounding effects of GVHD, we can significantly improve the predictive validity of preclinical trials, obtain broadly applicable findings, improve the efficiency of drugs, enhance safety profiling, and develop better therapeutic strategies. This approach is crucial in ensuring that the immunotherapeutic strategies developed in the laboratory are reflective of the human physiological response, thereby bridging a critical translational gap in oncological research.

## Introduction

 In the past few years, there has been an increasing demand to comprehend the connections between cancer progression and the immune system, leading to the initiation of fresh investigations. Notably, endeavors to stimulate the immune system and devise corresponding treatments to attain potent antitumor reactions have gained momentum.^1^

 The utilization of cancer immunotherapy as a novel therapeutic approach for the management of diverse malignancies has garnered noteworthy attention. This article outlines a variety of therapeutic strategies, encompassing immune checkpoint inhibitors (ICI), adoptive cell transfer (ACT), and cancer vaccines.^2^ The use of animal models, particularly murine models, has played a critical role in the progression and evaluation of cancer immunotherapies. These models have provided crucial insights into the mechanisms of action and potential negative consequences of the mentioned treatments.^3^

 Graft-versus-host disease (GVHD) is a major complication associated with allogeneic hematopoietic stem cell transplantation. The aforementioned complexity presents a significant confounding factor in animal models utilized for cancer immunotherapy, thereby acting as a variable that can potentially distort results. GVHD is a pathological condition characterized by the identification and subsequent attack of host tissues by immune cells derived from the donor. This leads to inflammation and the damage to multiple organs.^4^ The presence of GVHD in animal models has the potential to pose challenges in the evaluation of immunotherapy outcomes, as it may contribute to the observed anti-tumor effects or cause unintended toxicities.^5^

 Thus, effectively addressing GVHD in research is not just a methodological consideration but a strategic imperative that could significantly shape the future of cancer treatment. GVHD can significantly impact the interpretation of immunotherapy outcomes in preclinical studies, potentially obscuring the true efficacy and safety of novel treatments. By developing strategies to minimize or eliminate GVHD, researchers can ensure that the results obtained from animal models are more accurately reflective of the potential human response. This is essential for the progression of immunotherapies from the laboratory to clinical trials, ultimately leading to the development of more effective and safer cancer treatments.

 The objective of this systematic review is to evaluate the existing literature pertaining to the confounding role of GVHD in animal models of cancer immunotherapy.

 The focus of the review will be on diverse categories of immunotherapies and the associated challenges. These include checkpoint inhibitors, which block proteins that prevent the immune system from attacking cancer cells, chimeric antigen receptor (CAR) T-cell therapy, involving genetically engineered T-cells to better recognize and attack cancer cells, and cancer vaccines designed to elicit an immune response against specific cancer antigens. The other categories are oncolytic viruses that selectively infect and destroy tumor cells while stimulating an immune response and ACT, which boosts natural cancer-fighting cells in the immune system. Each of these therapies has shown promise in preclinical studies, utilizing a range of animal models to investigate their efficacy, safety, and mechanisms of action, paving the way for clinical trials and the development of new cancer treatments.

 Moreover, this paper examines prospective approaches addressing GVHD in animal models in order to enhance the reliability and precision of preclinical research on cancer immunotherapy.

## Materials and Methods

###  Search Strategy

 A comprehensive literature search was conducted in PubMed, Scopus, Embase, and Web of Science databases using several search terms, such as “cancer immunotherapy”, “animal models”, “graft-versus-host disease”, “GVHD”, and “confounding role”. Original research articles and reviews published in English from January 2000 to September 2023 were included in this review. However, studies were excluded if they focused solely on human subjects or in vitro models and were not published in English. Editorials, opinion articles, case reports, and case series were excluded as well. Two independent reviewers screened titles and abstracts for eligibility, and any discrepancies were resolved through discussion. This systematic review has been registered on PROSPERO (ID number CRD42024488544).

###  Data Extraction and Quality Assessment

 The data were extracted independently by two reviewers. For each included study, we extracted the following information: name of the author, year of publication, animal model, cancer type, immunotherapy type, GVHD impact on efficacy and safety, strategies to mitigate GVHD, inclusion and exclusion criteria, advantages, limitations, and key findings, for each study.

 The methodological quality of each study was assessed using the Systematic Review Centre for Laboratory Animal Experimentation (SYRCLE).

###  Risk of Bias Assessment

 The animal study design and reporting were evaluated by employing the SYRCLE tool, which aims to identify and disclose any potential bias in the animal studies that were included in the analysis.^6^

## Results

**Figure 1:**
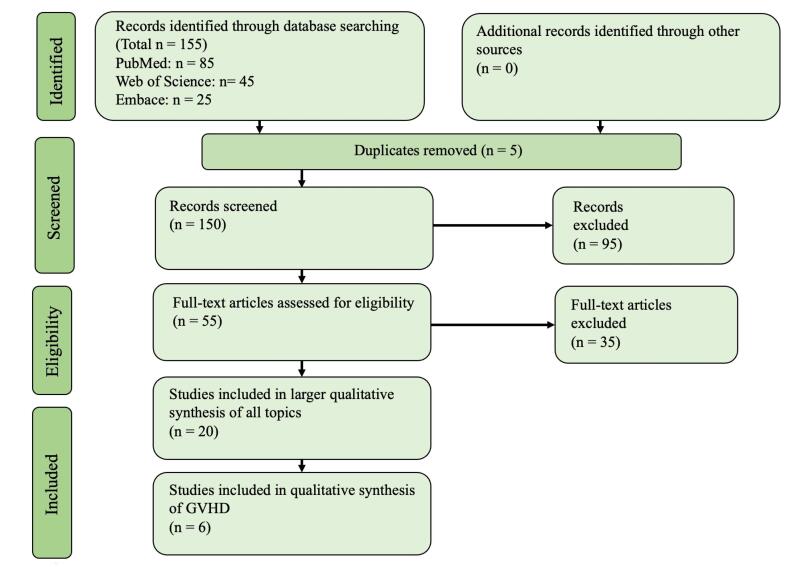
The PRISMA Diagram Illustrating the Process of Identifying the Articles Included in the Analysis. *Note*. PRISMA: Preferred Reporting Items for Systematic Reviews and Meta-Analyses

 A total of 155 articles were obtained from our search. Following a thorough evaluation process, 6 articles were included in the final assessment (Figure 1).

 GVHD is a significant confounding factor in preclinical models of cancer immunotherapy, which has the capacity to influence the analysis of empirical outcomes.^7^

 The implementation of techniques to tackle GVHD in the animal models of cancer immunotherapy encompasses the utilization of syngeneic or humanized mouse models, which may reduce the likelihood of alloreactivity, and the creation of gene-edited universal immune cells that manifest decreased alloreactivity.^8,9^

 Through the implementation of these methodologies, scholars can enhance the precision and dependability of preclinical investigations on cancer immunotherapy, resulting in the development of more efficacious and secure therapies for individuals.

 The main findings of the systematic review, including the types of immunotherapy, the animal models used, the incidence of GVHD, and strategies to minimize GVHD in the respective models, and the key references for each immunotherapy type are provided in Table 1.

**Table 1:** The Main Findings of the Systematic Review, Including Immunotherapy, the Applied Animal Models, the Incidence of GVHD, Strategies to Minimize GVHD in Respective Models, and Key References for Each Immunotherapy Type

**Immunotherapy Type**	**Animal Models**	**GVHD Incidence**	**Strategies to Minimize GVHD**	**References**
Immune checkpoint inhibitors	Humanized mice and syngeneic mice	Moderate	Humanized mouse models, careful selection of immune checkpoint targets	^10-13^
Adoptive cell transfer	Murine models and humanized mice	High	Syngeneic or humanized mouse models, gene-edited universal immune cells	^14-22^
Cancer vaccines	Murine models and humanized mice	Moderate to High	Syngeneic or autologous DCs, alternative antigen-presenting cells, and humanized mouse models	^23-29^

*Note*. GVHD: Graft-versus-host disease; DC: Dendritic cells.

 The present systematic review elucidates the difficulties pertaining to GVHD in various kinds of cancer immunotherapies and accentuates the significance of carefully designed animal investigations to mitigate the effects of GVHD.

###  Risk of Bias Evaluation

 The SYRCLE tool was utilized to assess the bias risk in this pre-clinical animal study (Table 2).^6^ Three out of the six studies provided baseline characteristics of the mice. However, the initial number of mice who received transplants and the sample size calculations were not clearly stated in all of the studies. Four out of the six studies defined random allocation of animals in control and experimental groups. Additionally, two studies mentioned blinding through randomization or outcome assessments. It was not evident that there was any attrition bias in any of the studies, and all treated animals were included in the final assessment. Table 3 presents the assigned risk of bias for each statement in some of the main studies.

**Table 2:** The Application of the SYRCLE Tool for Risk of Bias in Preclinical Studies

**Study**	**Selection Bias**			**Performance Bias**		**Detection Bias**		**Attrition Bias**	**Reporting Bias**	**Other**
	**Sequence Generation**	**Baseline Characteristics**	**Allocation Concealment**	**Random Housing**	**Blinding**	** Random Outcome Assessment**	**Blinding**	**Incomplete Outcome Data**	**Selective Outcome Reporting**	**Other Sources Of Bias**
Yan et al^30^	**○**	**○**	**○**	**○**	**●**	**○**	**●**	**○**	**○**	**○**
Kanikarla Marie et al^22^	**○**	**○**	**○**	**●**	**●**	**○**	**●**	**○**	**○**	**○**
Xing et al^31^	**○**	**●**	**○**	**○**	**●**	**○**	**●**	**○**	**○**	**○**
Riesner et al^8^	**○**	**●**	**○**	**○**	**○**	**○**	**○**	**○**	**○**	**○**
Poirot et al^32^	**○**	**○**	**○**	**○**	**●**	**○**	**●**	**○**	**○**	**○**
Koyama et al^33^	**○**	**●**	**○**	**●**	**○**	**○**	**○**	**○**	**○**	**○**

*Note*. SYRCLE: Systematic Review Centre for Laboratory Animal Experimentation. Each study is depicted with different risk levels, including unclear risk (red circles), low risk (white circles), and high risk (black circles).

**Table 3:** Summary of Main Findings

**Aspects**	**Main Findings**
1. Impact of GVHD on efficacy and safety of cancer immunotherapy	- GVHD can enhance or attenuate antitumor responses, thus affecting the efficacy of cancer immunotherapies.^4,33^ - GvHD-associated toxicities can confound the evaluation of immunotherapy-related adverse events.^30,34^
2. Limitations and challenges in interpreting the results of animal studies	- GVHD can obscure the assessment of immunotherapy efficacy and safety.^4^ - Variability in GVHD incidence and severity across different animal models complicates the interpretation of study results.^35^ - Animal models may not always accurately recapitulate the human immune system and tumor biology, limiting the translatability of findings.^36^
3. Strategies to mitigate GVHD in animal models	- Use of syngeneic models to reduce the risk of GVHD.^8^ - Administration of immunosuppressive agents to prevent or treat GvHD.^37^ - Gene editing technologies, such as CRISPR/Cas9, to modify donor immune cells and reduce alloreactivity.^32^
4. Insights into future research directions	- Development of animal models with reduced GVHD incidence and severity, and better representation of the human immune system and tumor biology.^36^ - Improved understanding of the mechanisms underlying GVHD and its impact on the efficacy and safety of cancer immunotherapy.^4,38^ - Implementation of more rigorous study designs and reporting standards in preclinical studies.^35^

*Note*. GVHD: Graft-versus-host disease.

###  Graft-Versus-Host Disease in Animal Models of Immune Checkpoint Inhibitors

 ICIs are utilized to enhance anti-tumor immune responses by targeting inhibitory receptors found on immune cells, including cytotoxic T-lymphocyte-associated protein 4 (CTLA-4) and programmed cell death protein 1 (PD-1).^39^ The utilization of animal models has played a crucial role in comprehending the mechanisms that underlie the anti-tumor impacts of ICIs and predicting their clinical efficacy.^10^ The emergence of GVHD in these models may interfere with the comprehension of ICI efficiency and adverse effects.^34^

 Several studies have reported the occurrence of GVHD in murine models of ICIs, specifically in the setting of allogeneic hematopoietic stem cell transplantation.^11,12^ Blazar et al conducted a study that showed how CTLA-4 blockade led to increased severity of GVHD in a murine model, thereby making it difficult to evaluate anti-tumor responses.^13^ The aforementioned findings emphasize the necessity of performing carefully planned animal studies that consider the probable confounding impacts of GVHD.

###  Graft-Versus-Host Disease in Animal Models of Adoptive Cell Transfer

 ACT entails the manipulation and ex vivo expansion of immune cells, including CAR^6^ T cells or tumor-infiltrating lymphocytes (TILs), which are subsequently administered to patients to elicit anti-tumor responses.^40^ The utilization of animal models has been of utmost importance in the advancement and refinement of ACT therapies. However, the issue of GVHD continues to pose a significant challenge in these models.

 GVHD has been observed in various animal models of ACT, particularly in the context of allogeneic T-cell transfer.^14,15^ For example, a study by Berger et al demonstrated that the transfer of allogeneic TILs led to GVHD in a murine model, confounding the assessment of the anti-tumor effects of ACT.^16^ Similar issues have been reported in animal models of CAR T cell therapy, where GVHD can arise from the use of allogeneic T cells or the recognition of normal tissues by the infused CAR T cells.^17,18^

 One strategy to minimize GVHD in animal models of ACT is the use of syngeneic or humanized mouse models, which can reduce the risk of alloreactivity.^19,20^ Additionally, the development of “off-the-shelf” universal CAR T cells that have been gene-edited to reduce alloreactivity may help address the issue of GVHD in preclinical models.^21^

###  Graft-Versus-Host Disease in Animal Models of Cancer Vaccines

 Cancer vaccines aim to stimulate the immune system to recognize and eliminate tumor cells by presenting tumor-associated antigens to immune cells.^23^ Animal models have been critical in the development and evaluation of cancer vaccines, but GVHD remains a potential confounding factor.

 GVHD has been reported in animal models of cancer vaccines, particularly when using allogeneic dendritic cells (DCs) as antigen-presenting cells.^24,25^ For instance, a study by Lutz et al revealed that the administration of allogeneic DCs resulted in GVHD in a murine model, complicating the interpretation of vaccine efficacy and safety.^26^

 To minimize the risk of GVHD in animal models of cancer vaccines, researchers can use syngeneic or autologous DCs to present tumor antigens or employ alternative antigen-presenting cells, such as macrophages or B cells.^27,28^ Additionally, the use of humanized mouse models may help assess the safety and efficacy of cancer vaccines more accurately in the absence of GVHD.^29^

###  Impact of Graft-Versus-Host Disease on Cancer Immunotherapy Efficacy and Safety

 GVHD has the potential to impact the effectiveness of cancer immunotherapies by modifying the interactions between immune cells and the tumor microenvironment.^4^ In a mouse model of melanoma, for instance, GVHD was found to boost the anticancer effects of ICIs.^30,41^ Nevertheless, divergent outcomes have been documented in alternative research, whereby GVHD has resulted in diminished anti-neoplastic potency in some cases.^33^

 Yan et al indicated that it is crucial to begin by establishing patient-derived xenograft (PDX) tumors alongside human immune cells to ensure precise immune editing. Subsequently, PDX tumors can be expanded into a larger group of humanized mice to assess therapy response. The potential for toxicity and GVHD is significantly reduced by following this approach. Moreover, this methodology enables the conduction of preclinical studies involving the combination of ICIs and targeted therapies. The findings from such studies can then be utilized to inform clinical trials and determine patient responses to therapy.^30^

 GVHD has been observed to have an impact on the safety of cancer immunotherapies, resulting in elevated morbidity and mortality rates in animal models.^5^ The interpretation of immunotherapy-related adverse events can be complicated by GVHD-associated toxicities, which include immune dysregulation, inflammation, and organ damage.^34^ The determination of the true safety profile of cancer immunotherapies is a challenging task in preclinical studies.

## Discussion

###  Limitations and Challenges in Interpreting the Results of Animal Studies

 The occurrence of GVHD in animal models presents several limitations and difficulties in the interpretation of outcomes from studies into cancer immunotherapy. GVHD has the potential to complicate the evaluation of immunotherapy effectiveness due to its influence on the tumor microenvironment and immune cell interactions, which can either augment or diminish the anti-tumor response.^4,33^ The evaluation of immunotherapy-related adverse events can be complicated by GVHD-associated toxicities, thereby hindering the determination of the actual safety profile of these therapies.^41^ The interpretation of study results is complicated by the variability in GVHD incidence and severity observed in different animal models, donor-host combinations, and treatment regimens.^35^

 In addition, it should be noted that the fidelity of the human immune system and tumor biology may not always be faithfully replicated by animal models, thereby constraining the applicability of research outcomes to clinical contexts.^36^ The utilization of mouse models that have been humanized through the engraftment of human immune cells and tumors represents a potential solution to this constraint. However, it is important to note that these models remain vulnerable to GVHD, as per previous research.^42^

 Xing et al revealed that the occurrence of GVHD in the MyD88–/– mouse is contingent upon the activity and growth of cells derived from the donor, specifically CD11c + DCs, in the target organs affected by GVHD. These findings highlight a novel function of host MyD88 in safeguarding against GVHD following allogeneic bone marrow transplantation.^31^

###  Strategies to Mitigate Graft-Versus-Host Disease in Animal Models

 In animal models of cancer immunotherapy, several techniques have been suggested to reduce GVHD. One approach involves the utilization of syngeneic models, wherein the donor and recipient animals are genetically identical, thereby minimizing the likelihood of GVHD.^43,44^ Nonetheless, this approach may not be appropriate for all categories of immunotherapeutic interventions, particularly those that are dependent on allogeneic immune responses.

 An additional approach involves the use of immunosuppressive agents for the prevention or treatment of GVHD.^37^ The administration of these agents may vary depending on the severity and location of GVHD, with systemic or local routes being considered. Although this method has demonstrated efficacy in mitigating the severity of GVHD, it has the potential to impede the antitumor effectiveness of immunotherapies due to the immunosuppressive properties of the agents, which can suppress immune responses.^22^

 The application of gene editing technologies, such as CRISPR/Cas9, has been suggested as a strategy to alleviate GVHD by altering the immune cells of the donor to decrease their alloreactivity.^32^ The aforementioned methodology has demonstrated potential in preclinical investigations; however, additional inquiry is required to ascertain its viability and safety in clinical environments.

Table 4 provides a summary of selected studies investigating the confounding role of GVHD in animal models of cancer immunotherapy. The studies cover various cancer types, immunotherapy types, and strategies to mitigate GVHD. The numbers in brackets refer to the corresponding references mentioned earlier in the text. Further, Table 5 summarizes the different types of animal models used in cancer studies, including their description, advantages, and limitations, and Table 3 describes the main findings.

**Table 4:** A Summary of Selected Studies Investigating the Confounding Role of GVHD in Animal Models of Cancer Immunotherapy

**Study**	**Year**	**Animal Model**	**Cancer Type**	**Immunotherapy Type**	**GVHD Impact on Efficacy**	**GVHD Impact on Safety**	**Strategies to Mitigate GVHD**
Yan et al^30^	2023	Mouse	Melanoma	Immune checkpoint blockade	Enhanced antitumor effects	Increased toxicities	Not reported
Kanikarla Marie et al^22^	2022	Mouse	Colorectal Cancer	T cells	Tumor volume reduction	Delay the impact of GVHD	Immunosuppressive agents
Xing et al^31^	2019	Mouse	Various	Allogeneic bone marrow transplantation	Not reported	Reduced GVHD severity	Immunosuppressive agents
Riesner et al^8^	2016	Mouse	Various	Allogeneic hematopoietic stem cell transplantation	Not reported	Reduced GVHD	Immunosuppressive agents
Poirot et al^32^	2015	Mouse	B-cell leukemia	CAR T cells	Not reported	Reduced GVHD incidence	CRISPR/*Cas9* gene editing
Koyama et al^33^	2011	Mouse	Various	Allogeneic hematopoietic stem cell transplantation	Reduced antitumor efficacy	Increased morbidity and mortality	Not reported

*Note*. GVHD: Graft-versus-host disease.

**Table 5:** Summary of Selected Studies Investigating the Confounding Role of GVHD in Animal Models of Cancer Immunotherapy

**Study**	**Year**	**Animal Model**	**Cancer Type**	**Immunotherapy Type**	**GVHD Impact on Efficacy**	**GVHD Impact on Safety**	**Strategies to Mitigate GVHD**
Yan et al^30^	2023	Mouse	Melanoma	Immune checkpoint blockade	Enhanced antitumor effects	Increased toxicities	Not reported
Kanikarla Marie et al^22^	2022	Mouse	Colorectal cancer	T cells	Tumor volume reduction	Delay the impact of GVHD	Immunosuppressive agents
Xing et al^31^	2019	Mouse	Various	Allogeneic bone marrow transplantation	Not reported	Reduced GVHD severity	Immunosuppressive agents
Riesner et al^8^	2016	Mouse	Various	Allogeneic hematopoietic stem cell transplantation	Not reported	Reduced GVHD	Immunosuppressive agents
Poirot et al^32^	2015	Mouse	B-cell leukemia	CAR T cells	Not reported	Reduced GVHD incidence	CRISPR/*Cas9* gene editing
Koyama et al^33^	2011	Mouse	Various	Allogeneic hematopoietic stem cell transplantation	Reduced antitumor efficacy	Increased morbidity and mortality	Not reported

*Note*. GVHD: Graft-versus-host disease.

###  Immunodeficient and Humanized Mouse Models

 Immunodeficient and humanized mouse models have played a pivotal role in advancing our understanding of cancer immunotherapy, particularly in circumventing the challenges posed by xenogeneic GVHD. The development of these models has undergone several evolutionary stages, each marked by significant improvements in mimicking human immune responses.

 The first generation of immunodeficient mice, the nude mice, lacking thymus and therefore T cells, were a significant step but had limitations due to residual immune activity.^45^ This led to the development of severe combined immunodeficiency (SCID) mice, which lacked both B and T cells, offering a better platform for cancer studies.^46^ However, the SCID mice still presented limitations, particularly in the context of innate immunity. This issue was partially addressed with the advent of non-obese diabetic SCID (NOD-SCID) mice, which showed further impairment in natural killer (NK) cell function, enhancing their utility in xenograft studies.^47^

 The next significant advancement was the NOD-SCID gamma (NSG) mice, which, due to a mutation in the *interleukin* 2 receptor gamma chain, exhibited a near-complete absence of functional immune cells.^48^ These mice became the gold standard for humanized models, allowing for the engraftment of human cells and tissues without significant xenogeneic GVHD, thus providing a more accurate representation of human immune responses in cancer immunotherapy research.^49^

 Finally, the development of fully humanized mice, achieved through the engraftment of human hematopoietic stem cells into immunodeficient hosts, marked a critical juncture. These models closely mimic the human immune system, allowing for a more precise study of human-specific immune responses to cancer and therapies while significantly reducing the confounding effects of xenogeneic GVHD.^50^

 This evolutionary trajectory of mouse models has been fundamental into cancer immunotherapy research. Each advancement has progressively bridged the gap between preclinical studies and clinical applicability, enhancing our understanding of therapeutic mechanisms and improving the safety and efficacy of immunotherapeutic strategies.^51^

 Immunodeficient and humanized mouse models, despite their extensive use in cancer immunotherapy research, cannot entirely eliminate the risk of GVHD. This limitation is due to several inherent characteristics of these models and the complex nature of human immune cell engraftment.

 Firstly, even though immunodeficient mice such as the NSG (NOD-scid IL2Rγ^null^) model lack functional B, T, and NK cells, the introduction of human immune cells can lead to GVHD due to the human cells recognizing mouse tissues as foreign. This is because minor histocompatibility antigens still present in these mice can be targeted by the engrafted human immune cells.^47^

 Secondly, humanized mouse models, created by engrafting human hematopoietic stem cells into immunodeficient mice, are designed to closely mimic the human immune system. While these models allow for the study of human-specific immune responses, the mismatch between the human immune cells and the mouse tissue antigens can lead to the development of GVHD.^48^ This is compounded by the fact that the reconstitution of the immune system in these mice is not always perfectly balanced, leading to an environment where GVHD can develop.^52^

 Furthermore, the severity of GVHD in humanized mice can vary based on the strain of the mouse, the source and type of human cells used for engraftment, and the conditioning regimen applied to the mice before engraftment.^53^ These variables can affect the incidence and severity of GVHD, making it a persistent risk in these models.

 To mitigate the risk of GVHD, researchers have explored various strategies, including genetic modifications to create mouse models that better tolerate human cells and the use of immunosuppressive drugs post-engraftment. However, these strategies often involve a trade-off between reducing GVHD and preserving the functionality of the humanized immune system for research purposes.^54^

## Insights into Future Research Directions

1. *Exploring novel animal models:* Continued development and refinement of animal models that either inherently resist the development of GVHD or can be easily manipulated to study cancer immunotherapy without the confounding effects of GVHD. This includes the exploration of alternative species or strains with different immune system compatibilities.^55^
2. *Advancing gene-editing technologies:* Leveraging CRISPR-Cas9 and other gene-editing tools to create more sophisticated immunodeficient and humanized mouse models. These advancements can help in precisely knocking out or modifying genes responsible for GVHD initiation, allowing for a clearer assessment of immunotherapies.^56^
3. *Focusing on standardized assessment and reporting of graft-versus-host disease*:Establishing universal guidelines for the assessment, grading, and reporting of GVHD in animal studies. Standardization would facilitate more accurate comparisons between studies and provide a clearer understanding of the GVHD impact on cancer immunotherapy outcomes.^57^
4. *Utilizing immunomodulatory strategies:*Investigating the use of immunomodulatory drugs or cellular therapies to prevent or treat GVHD without compromising the immune response against the tumor. This could include the use of regulatory T cells, cytokine blockers, or checkpoint inhibitors, specifically targeting the pathways involved in GVHD.^58^
5. *Incorporating humanized immune system components:* Beyond full human immune system reconstitution, focusing on humanizing specific components of the mouse immune system relevant to the immunotherapy being tested. This approach may reduce GVHD while still providing insights into the human immune response.^50^
6. *Developing predictive biomarkers for graft-versus-host disease:* Identifying and validating biomarkers that can predict the onset or severity of GVHD in animal models. Such biomarkers could help in early intervention and more precise monitoring of GVHD, improving the reliability of cancer immunotherapy studies.^57^

## Conclusion

 GVHD represents a confounding factor in preclinical cancer immunotherapy models, which can significantly affect the accurate assessment of both safety and efficacy endpoints. The creation of animal models that exhibit decreased incidence and severity of GVHD, coupled with a deeper comprehension of the underlying mechanisms, can serve as a means to overcome these obstacles and promote the progress of secure and efficacious cancer immunotherapies.

## References

[1] Kum Özşengezer S, Altun Z (2023). Animal models for cancer immunology. Curr Mol Biol Rep.

[2] Topalian SL, Drake CG, Pardoll DM (2015). Immune checkpoint blockade: a common denominator approach to cancer therapy. Cancer Cell.

[3] Day CP, Merlino G, Van Dyke T (2015). Preclinical mouse cancer models: a maze of opportunities and challenges. Cell.

[4] Zeiser R, Blazar BR (2017). Acute graft-versus-host disease - biologic process, prevention, and therapy. N Engl J Med.

[5] Ferrara JL, Levine JE, Reddy P, Holler E (2009). Graft-versus-host disease. Lancet.

[6] Hooijmans CR, Rovers MM, de Vries RB, Leenaars M, Ritskes-Hoitinga M, Langendam MW (2014). SYRCLE’s risk of bias tool for animal studies. BMC Med Res Methodol.

[7] Elhage A, Sligar C, Cuthbertson P, Watson D, Sluyter R (2022). Insights into mechanisms of graft-versus-host disease through humanised mouse models. Biosci Rep.

[8] Riesner K, Kalupa M, Shi Y, Elezkurtaj S, Penack O (2016). A preclinical acute GVHD mouse model based on chemotherapy conditioning and MHC-matched transplantation. Bone Marrow Transplant.

[9] Murty T, Mackall CL (2021). Gene editing to enhance the efficacy of cancer cell therapies. Mol Ther.

[10] De La Rochere P, Guil-Luna S, Decaudin D, Azar G, Sidhu SS, Piaggio E (2018). Humanized mice for the study of immuno-oncology. Trends Immunol.

[11] Yang Y, Shaffer AL 3rd, Emre NC, Ceribelli M, Zhang M, Wright G (2012). Exploiting synthetic lethality for the therapy of ABC diffuse large B cell lymphoma. Cancer Cell.

[12] Davids MS, Kim HT, Bachireddy P, Costello C, Liguori R, Savell A (2016). Ipilimumab for patients with relapse after allogeneic transplantation. N Engl J Med.

[13] Blazar BR, Carreno BM, Panoskaltsis-Mortari A, Carter L, Iwai Y, Yagita H (2003). Blockade of programmed death-1 engagement accelerates graft-versus-host disease lethality by an IFN-gamma-dependent mechanism. J Immunol.

[14] Bleakley M, Heimfeld S, Jones LA, Turtle C, Krause D, Riddell SR (2014). Engineering human peripheral blood stem cell grafts that are depleted of naïve T cells and retain functional pathogen-specific memory T cells. Biol Blood Marrow Transplant.

[15] Riddell SR, Watanabe KS, Goodrich JM, Li CR, Agha ME, Greenberg PD (1992). Restoration of viral immunity in immunodeficient humans by the adoptive transfer of T cell clones. Science.

[16] Berger C, Jensen MC, Lansdorp PM, Gough M, Elliott C, Riddell SR (2008). Adoptive transfer of effector CD8 + T cells derived from central memory cells establishes persistent T cell memory in primates. J Clin Invest.

[17] Kochenderfer JN, Dudley ME, Feldman SA, Wilson WH, Spaner DE, Maric I (2012). B-cell depletion and remissions of malignancy along with cytokine-associated toxicity in a clinical trial of anti-CD19 chimeric-antigen-receptor-transduced T cells. Blood.

[18] Eyquem J, Mansilla-Soto J, Giavridis T, van der Stegen SJ, Hamieh M, Cunanan KM (2017). Targeting a CAR to the TRAC locus with CRISPR/Cas9 enhances tumour rejection. Nature.

[19] Shultz LD, Goodwin N, Ishikawa F, Hosur V, Lyons BL, Greiner DL (2014). Human cancer growth and therapy in immunodeficient mouse models. Cold Spring Harb Protoc.

[20] Mosier DE, Gulizia RJ, Baird SM, Wilson DB (1988). Transfer of a functional human immune system to mice with severe combined immunodeficiency. Nature.

[21] Qasim W, Zhan H, Samarasinghe S, Adams S, Amrolia P, Stafford S (2017). Molecular remission of infant B-ALL after infusion of universal TALEN gene-edited CAR T cells. Sci Transl Med.

[22] Kanikarla Marie P, Sorokin AV, Bitner LA, Aden R, Lam M, Manyam G (2022). Autologous humanized mouse models to study combination and single-agent immunotherapy for colorectal cancer patient-derived xenografts. Front Oncol.

[23] Finn OJ (2008). Cancer immunology. N Engl J Med.

[24] Dhodapkar MV, Steinman RM, Krasovsky J, Munz C, Bhardwaj N (2001). Antigen-specific inhibition of effector T cell function in humans after injection of immature dendritic cells. J Exp Med.

[25] Morse MA, Coleman RE, Akabani G, Niehaus N, Coleman D, Lyerly HK (1999). Migration of human dendritic cells after injection in patients with metastatic malignancies. Cancer Res.

[26] Lutz E, Yeo CJ, Lillemoe KD, Biedrzycki B, Kobrin B, Herman J (2011). A lethally irradiated allogeneic granulocyte-macrophage colony stimulating factor-secreting tumor vaccine for pancreatic adenocarcinoma: a phase II trial of safety, efficacy, and immune activation. Ann Surg.

[27] Palucka K, Banchereau J (2012). Cancer immunotherapy via dendritic cells. Nat Rev Cancer.

[28] Bol KF, Schreibelt G, Gerritsen WR, de Vries IJ, Figdor CG (2016). Dendritic cell-based immunotherapy: state of the art and beyond. Clin Cancer Res.

[29] Ito R, Takahashi T, Katano I, Ito M (2012). Current advances in humanized mouse models. Cell Mol Immunol.

[30] Yan C, Nebhan CA, Saleh N, Shattuck-Brandt R, Chen SC, Ayers GD (2023). Generation of orthotopic patient-derived xenografts in humanized mice for evaluation of emerging targeted therapies and immunotherapy combinations for melanoma. Cancers (Basel).

[31] Xing S, Zhang X, Liu JH, Huang X, Zhou P (2019). Host MyD88 signaling protects against acute graft-versus-host disease after allogeneic bone marrow transplantation. Clin Exp Immunol.

[32] Poirot L, Philip B, Schiffer-Mannioui C, Le Clerre D, Chion-Sotinel I, Derniame S (2015). Multiplex genome-edited T-cell manufacturing platform for “off-the-shelf” adoptive T-cell immunotherapies. Cancer Res.

[33] Koyama M, Kuns RD, Olver SD, Raffelt NC, Wilson YA, Don AL (2011). Recipient nonhematopoietic antigen-presenting cells are sufficient to induce lethal acute graft-versus-host disease. Nat Med.

[34] Nguyen LS, Raia L, Lebrun-Vignes B, Salem JE (2020). Graft versus host disease associated with immune checkpoint inhibitors: a pharmacovigilance study and systematic literature review. Front Pharmacol.

[35] Shono Y, van den Brink MR (2018). Gut microbiota injury in allogeneic haematopoietic stem cell transplantation. Nat Rev Cancer.

[36] Ruggeri L, Capanni M, Urbani E, Perruccio K, Shlomchik WD, Tosti A (2002). Effectiveness of donor natural killer cell alloreactivity in mismatched hematopoietic transplants. Science.

[37] Korngold R, Sprent J (1978). Lethal graft-versus-host disease after bone marrow transplantation across minor histocompatibility barriers in mice Prevention by removing mature T cells from marrow. J Exp Med.

[38] Pidala J, Walton K, Elmariah H, Kim J, Mishra A, Bejanyan N (2020). Biological and clinical impact of JAK2/mTOR blockade in GVHD prevention: preclinical and phase I trial results. Blood.

[39] Sharma P, Allison JP (2015). Immune checkpoint targeting in cancer therapy: toward combination strategies with curative potential. Cell.

[40] Rosenberg SA, Restifo NP (2015). Adoptive cell transfer as personalized immunotherapy for human cancer. Science.

[41] Zitvogel L, Apetoh L, Ghiringhelli F, Kroemer G (2008). Immunological aspects of cancer chemotherapy. Nat Rev Immunol.

[42] Walsh NC, Kenney LL, Jangalwe S, Aryee KE, Greiner DL, Brehm MA (2017). Humanized mouse models of clinical disease. Annu Rev Pathol.

[43] Cogels MM, Rouas R, Ghanem GE, Martinive P, Awada A, Van Gestel D (2021). Humanized mice as a valuable pre-clinical model for cancer immunotherapy research. Front Oncol.

[44] Zeng Z, Wong CJ, Yang L, Ouardaoui N, Li D, Zhang W (2022). TISMO: syngeneic mouse tumor database to model tumor immunity and immunotherapy response. Nucleic Acids Res.

[45] Flanagan SP (1966). ‘Nude’, a new hairless gene with pleiotropic effects in the mouse. Genet Res.

[46] Bosma GC, Custer RP, Bosma MJ (1983). A severe combined immunodeficiency mutation in the mouse. Nature.

[47] Shultz LD, Lyons BL, Burzenski LM, Gott B, Chen X, Chaleff S (2005). Human lymphoid and myeloid cell development in NOD/LtSz-SCID IL2R gamma null mice engrafted with mobilized human hemopoietic stem cells. J Immunol.

[48] Ito M, Hiramatsu H, Kobayashi K, Suzue K, Kawahata M, Hioki K (2002). NOD/SCID/gamma(c)(null) mouse: an excellent recipient mouse model for engraftment of human cells. Blood.

[49] Shultz LD, Brehm MA, Garcia-Martinez JV, Greiner DL (2012). Humanized mice for immune system investigation: progress, promise and challenges. Nat Rev Immunol.

[50] Wunderlich M, Chou FS, Link KA, Mizukawa B, Perry RL, Carroll M (2010). AML xenograft efficiency is significantly improved in NOD/SCID-IL2RG mice constitutively expressing human SCF, GM-CSF and IL-3. Leukemia.

[51] Drake AC, Chen Q, Chen J (2012). Engineering humanized mice for improved hematopoietic reconstitution. Cell Mol Immunol.

[52] King MA, Covassin L, Brehm MA, Racki W, Pearson T, Leif J (2009). Human peripheral blood leucocyte non-obese diabetic-severe combined immunodeficiency interleukin-2 receptor gamma chain gene mouse model of xenogeneic graft-versus-host-like disease and the role of host major histocompatibility complex. Clin Exp Immunol.

[53] Pearson T, Greiner DL, Shultz LD (2008). Creation of “humanized” mice to study human immunity. Curr Protoc Immunol.

[54] Theocharides AP, Rongvaux A, Fritsch K, Flavell RA, Manz MG (2016). Humanized hemato-lymphoid system mice. Haematologica.

[55] Shultz LD, Goodwin N, Ishikawa F, Hosur V, Lyons BL, Greiner DL (2014). Human cancer growth and therapy in immunodeficient mouse models. Cold Spring Harb Protoc.

[56] Platt RJ, Chen S, Zhou Y, Yim MJ, Swiech L, Kempton HR (2014). CRISPR-Cas9 knockin mice for genome editing and cancer modeling. Cell.

[57] Cooke KR, Luznik L, Sarantopoulos S, Hakim FT, Jagasia M, Fowler DH (2017). The biology of chronic graft-versus-host disease: a task force report from the National Institutes of Health Consensus Development Project on Criteria for Clinical Trials in Chronic Graft-versus-Host Disease. Biol Blood Marrow Transplant.

[58] Blazar BR, MacDonald KPA, Hill GR (2018). Immune regulatory cell infusion for graft-versus-host disease prevention and therapy. Blood.

